# Diseases of the Vascular System

**Published:** 1894-08-25

**Authors:** 


### Progress in Medicine.

### DISEASES OF THE HEART AND VASCULAR SYSTEM.

**Physics of the Circulation.**—In his recent Goulstonian lectures Dr. Paul Chapman has endeavoured to place on a clearer and firmer basis the graphic method, or rather the combined graphic method, in the investigation of the phenomena of the circulation in health and disease. This subject is a most difficult one to treat; in fact, so many issues are involved, and the sources of error are so numerous and variable, that the task of properly interpreting the data obtained only after elaborate care, has proved a matter of great difficulty to the most highly trained experts, and has given rise to discussions on many points which still require elucidation. These methods of clinical investigation have, therefore, most naturally failed to come into general use up to the present, but, beside placing before the present position of the graphic method, Dr. Chapman reminds us of the most recent contributions to cardiac physiology.

The first heart sound has been proved by Kasembek and others to be a muscle tone due to the ventricular systole, to which other sounds are superadded, especially those arising from the vibrations of the mitral and tricuspid valves, and also from the vibrations of the semilunar valves and to the vibrations of the columns of blood above them. With regard to the second sound, the lecturer observes that it will suffice to say that it disappears when the valves are destroyed, and that it consists of two sounds united, due respectively to the closure of the semilunar valves and to the vibrations of the blood columns above them.

As to the production of the apex beat, we have to consider the changes of position and of form of the heart during systole. An interesting experiment was performed by Haycraft[2], who noticed that if a needle is inserted through the chest wall into the heart, the end projecting from the thorax moves upward at each systole, while if the needle is passed through the thorax nearer to the apex the excursion of the needle is less. If it is inserted into the apex very little movement is communicated to the needle. As may be seen by direct inspection of the exposed heart, during systole the base approaches the apex, and the latter is the least moveable point of the heart.

That the common assumption that the pulmonary and aortic valves are closed by the regurgitation of blood against them owing to the relaxation of the ventricles and the consequent alteration in pressure on the two sides of the valves, is shown to be incorrect. During systole the blood is forced through the orifices, which at that time are mere chinks, owing to the pads of muscle which take their origin from all sides of the ostium. Vortices are thus created in the space between the arterial root and the edges of the valves. These vortices tend to press the edges of the valves together, and the valves consequently close the moment the blood actually ceases to stream through the narrow crevice. By this means all regurgitation is avoided, the valves being already closed before the recoil of the blood column against them.

There is a period, called by Keyt the "præsphymic" interval, during which the ventricular contraction is raising the intra-ventricular blood pressure to that in the aorta. Not till the intra-ventricular blood pressure exceeds that of the aorta can the aortic valves be opened, so that the emptying of the ventricle and the production of the pulse-wave follows the commencement of the systole by a short interval, and this is normally about one-tenth of the period of the whole cardiac cycle.

The semilunar valves must close when the aortic pressure exceeds that in the left ventricle. Whether the second sound is coincident with their closure is not made out; it may occur just subsequently to their closure by the vortices, the second sound being coincident with their tension, but not with their closure. By comparative estimation of the pressures in the ventricle and aorta, Von Frey has estimated the closure to take place in the upper half of the drop in the apex tracing; while Hürthle, and Chauveau, and Mary placed it a little higher and lower respectively.

Dr. Chapman shows that by far the largest part of the work of the heart is expended in overcoming the obstacles in the vascular system, while a very small part of the heart's work is available for imparting to the blood its requisite velocity. It is but one two-hundredth part of the whole work of the heart, and the importance of treasuring this small margin of force, and the readiness with which this blood flow can be extinguished by any increase in the vascular pressure, should be continually borne in mind.

While the force of ventricular contraction varies with the arterial pressure, the duration of the systole is independant of arterial or venous pressure. Dr. Chapman has met with very short systole in pernicious anæmia and in fatty heart. In these, as in all other cases, he considers that it is probable that marked alterations in the duration of the ventricular systole must be taken as evidence of want of nutrition, or of the presence in the blood of toxic substances. A rise or fall of pressure in the aorta will, on the other hand, affect the frequency of the pulse, which sinks by rise pressure, and rises by fall of pressure, through the influence of stimulation of the vagus centre or of the inhibitory nerves respectively.

As regards the obstacle offered by the capillaries to the wave of impulse sent through the blood, we must remember that the distance between the finest arterioles and the extreme venous radicles has been estimated at 0·2 m.m. only. It is now known that the capillary walls are contractile, and that the diameter of the capillaries varies independently of the arterial blood pressure. When they thus vary it is probably in response to the chemical constitution of the blood, which is altered in correspondence with the varying conditions of tissue change in the part. Irritation of the nerves which supply the capillaries has not yet been directly shown to influence their diameter, though variations in their diameter may assist the arterioles in regulating the blood supply to an organ.

The sphygmograph affords a means of observing variations in intra-arterial blood tension, but cannot in any way be taken as a measure of such tension. The sphygmogram presents certain definite points peculiar to all pulse curves. First, there is the steep rise, corresponding to a primary positive wave from the streaming of blood into the aorta. The slowness with which the rise takes place is some measure of the obstruction to the inflow of blood. After the summit of the primary wave is reached there is a drop to the dicrotic notch, following which there is a rise corresponding to the dicrotic wave. Between the summit of the first wave and the dicrotic notch there is an upheaval which is usually on the falling line between the first summit and the dicrotic notch. According to Roy and Adami, the upheaval corresponds to the continuance of outflow from the ventricle, and it is by them termed the "outflow remainder wave," and by others the "tidal" wave. The dicrotic notch is caused by a fall of pressure following on the first distension of the vessel, for the subsequent dicrotic upheaval is caused by a centrifugal positive wave; according to some, arising from the recoil of blood at the root of the aorta against the closed semilunar valves. There has been much discussion as to the exact origin of this dicrotic wave, and these are expressed and discussed by Dr. Chapman.

The lecturer explains the methods and the advantage of this combined method of registering the cardiac movements and the pulse tracings simultaneously, his observations being additionally interesting and valuable from the introduction of an ingenious chronograph. He has been able to show by these means that the cardio-radial interval should be about 0·17 of a second, and by accounting for the time occupied in the transmission of the pulse wave from the semilunar valves to the point of the carotid, whence a sphygmographic tracing was taken, and from the carotid to the radial, a period of ·07 of a second is left, which we must take to be the time occupied between the commencement of ventricular systole to the opening of the semilunar valves, this being the period termed by Keyt the præsphygmic interval. Speaking of this interval, Dr. Waller remarks: "This considerable delay affords a reason for the distinct presystolic character frequently recognisable as belonging to murmurs of aortic obstruction. It also explains why a first sound, heard undivided at the heart's apex is often as it were split into two portions if listened to above the clavicle, the first portion being the transmitted first sound, the second the arterial rush beneath the stethoscope."

The importance of recognising this præsphygmic interval is at once seen when we come to consider the conditions under which it is diminished or increased. Thus Dr. Chapman was able to diagnose aortic insufficiency in an emphysematous patient in whom all sounds at the base of the heart were entirely obscured by the wheezing and sibilus with loud rhonchi due to the lung conditions, but in whom the præsphygmic interval was greatly diminished. "Reflecting much over this case it occurred to me that, since the præsphygmic interval represented the time occupied in raising the intra-ventricular pressure, and that a pulse wave could not appear in the arteries until the semilunar valves were opened, there must in this case be some condition present whereby intra-ventricular pressure was equal to the aortic pressure at the commencement of systole. Such a condition would exist under the supposition that the semilunar valves were incomplete, whereby the aorta and ventricle formed one cavity. During diastole blood would freely regurgitate into the ventricle. At the end of diastole the intra-ventricular pressure would therefore be equal to that in the aorta, and at the commencement of systole a wave would straightway appear in the aorta without the intervention of a præsphygmic interval." Keyt has also demonstrated the shortening of this præsphygmic interval in aortic insufficiency. Further, Dr. Chapman claims that if we accept the foregoing arguments, "it follows that in cases where marked aortic insufficiency is known to exist, and there is either no alteration from the normal delay, or, still more, when there is an increase in the delay, we must assume the presence of some other retarding influence, of which the most obvious is aneurysm."

The mechanism of the cardiac movements has long been an unsettled question, and Romberg[3], in giving a review of the present state of knowledge on this point, states that active dilatation of the heart's cavities plays a principal rôle in the filling of the ventricles, as the regularly following phase of the systole shows a negative pressure. As regards the rhythmic contractions of the heart, it is now known that the old views that the cause resided in the cardiac ganglia alone is no longer tenable. It has been proved by His (junr.) and Romberg that these ganglia are of a sympathetic nature, and that the heart pulsates at a time at which it is still without ganglia; while Gaskell, Krehl, and Romberg have shown that the heart pulsates regularly even when it is deprived of ganglion cells. At most the vagus and accelerator may have a direct action on the muscular fibres. Heart poisons, such as atropine, muscarin, &c., injure not only through the ganglia, but also the heart muscle which is free from ganglia. From these observations the importance of the cardiac muscle must be placed in the foreground. It is in disturbed activity of the heart muscle that we must, in the first instance, seek for the key to the solution of the numerous deviations from the normal conditions of the circulation.

[1] Lancet, March 3, 10, and 17, 1894. [2] Journ. of Phys., 1893. [3] Berlin Klin. Woch., Nos. 12 and 13, 1893; Pract., Feb., 1894.

*(To be continued.)*

